# Management of adolescents with very poorly controlled type 1 diabetes by nurses: a parallel group randomized controlled trial

**DOI:** 10.1186/s13063-015-0923-7

**Published:** 2015-09-08

**Authors:** Behrouz Kassai, Muriel Rabilloud, Delphine Bernoux, Catherine Michal, Benjamin Riche, Tiphanie Ginhoux, Valérie Laudy, Daniel Terral, Catherine Didier-Wright, Veronique Maire, Catherine Dumont, Gilles Cottancin, Muriel Plasse, Guy-Patrick Jeannoel, Jamil Khoury, Claire Bony, Michel Lièvre, Jocelyne Drai, Marc Nicolino

**Affiliations:** EPICIME-CIC 1407 de Lyon, Hospices Civils de Lyon, Inserm, Service de Pharmacologie Clinique, F-69677 Bron, France; Université de Lyon, F-69000 Lyon, France; Hospices Civils de Lyon, Service de Biostatistiques, F-69324 Lyon, France; Endocrinologie, Diabétologie, Nutrition Pédiatriques, Hôpital Femme-Mère-Enfant, Hospices Civils de Lyon, F-69677 Bron, France; Service de Pédiatrie Générale Multidisciplinaire, Hôpital Estaing–CHU de Clermont Ferrand, F-63003 Clermont-Ferrand, France; Centre Hospitalier de la Région Annecienne (CHRA)–Service des Grands Enfants, F-74374 Pringy, France; Service de Pédiatrie, Hôpital de Vienne, F-38200 Vienne, France; Centre Hospitalier de Sallanches–Service de Pédiatrie, F-74700 Sallanches, France; Centre Hospitalier d’Albertville–Service de Pédiatrie, F-73200 Albertville Moûtiers, France; Service de Pédiatrie, Centre Hospitalier de Roanne, F-42300, Roanne, France; Service de Pédiatrie, Centre Hospitalier de Hyères, F-83407 Hyères, France; Cabinet de Pédiatrie, F-07100 Annonay, France; Fédération de Biochimie, Unité de Biochimie Métabolique et Moléculaire, Centre Hospitalier Lyon-Sud, F-69495 Pierre-Bénite, France; CNRS, UMR 5558, Laboratoire de Biométrie et Biologie Evolutive, Université Lyon 1, F-69622 Villeurbanne, France

**Keywords:** Randomized controlled trial, Diabetes mellitus, Type 1, Child, Adolescent

## Abstract

**Backgrounds:**

Fluctuation in glycemia due to hormonal changes, growth periods, physical activity, and emotions make diabetes management difficult during adolescence. Our objective was to show that a close control of patients’ self-management of diabetes by nurse-counseling could probably improve metabolic control in adolescents with type 1 diabetes.

**Methods:**

We designed a multicenter, randomized controlled, parallel group, clinical trial. Seventy seven adolescents aged 12–17 years with A1C >8 % were assigned to either an intervention group (pediatrician visit every 3 months + nurse visit and phone calls) or to the control group (pediatrician visit every 3 months). The primary outcome was the evolution of the rate of A1C during the 12 months of follow-up. Secondary outcomes include patient’s acceptance of the disease (evaluated by visual analog scale), the number of hypoglycemic or ketoacidosis episodes requiring hospitalization, and evaluation of A1C rate over time in each group.

**Results:**

Seventy-seven patients were enrolled by 10 clinical centers. Seventy (89.6 %) completed the study, the evolution of A1C and participants satisfaction over the follow-up period was not significantly influenced by the nurse intervention.

**Conclusion:**

Nurse-led intervention to improve A1C did not show a significant benefit in adolescents with type 1 diabetes because of lack of power. Only psychological management and continuous glucose monitoring have shown, so far, a slight but significant benefit on A1C. We did not show improvements in A1C control in teenagers by nurse-led intervention.

**Trial registration:**

Clinical Trials.gov registration number: NCT00308256, 28 March 2006.

## Background

Type 1 diabetes represents 10 to 15 % of all types of diabetes mellitus and 90 % of diabetes in children. In Europe the prevalence is estimated at 94,000 children below 15 years of age in 2005 with an increasing incidence ranging between 0.6 and 9.3 % [1]. In 2020, the incidence of diabetes is expected to double in children below 5 years of age [2, 3].

One of the main objectives in the management of type 1 diabetes is to educate patients in order to increase their autonomy and adherence to treatment [4–7]. Treatment adherence in young people seems more difficult to obtain than in adults, mainly because adolescents lack responsible self-management and deny their disease [8]. In France a survey of 446 young people with type 1 diabetes, aged 8–17 years who attended the 2009 summer camps, showed a mean glycated hemoglobin (A1C) varying from 7.9 to 8.4 %. Another survey suggests that the mean A1C was 9 % [9].

According to the International Society of Pediatric and Adolescent Diabetes [10] and American Diabetes Association [11], adolescents are a special group that requires specialized education and facilities to help to prevent serious long-term complications of diabetes including the drop-out of patients from clinic attendance and surveillance. Consequently, providing more friendly to use medicines, improving the well-being of children and adolescents, ensuring normal school and private life, and reducing psychological complications are the main goals of the management of type 1 diabetes.

Several non-pharmacological strategies comprising education, psychological management, diet and exercise have been evaluated for improving type 1 diabetes management in children and adolescents [12, 13]. We hypothesized that a stricter control of glycemia by nurse-counseling could probably improve metabolic control in adolescents. The main objective of our study was to show that nurse-counseling may improve levels of A1C and patient satisfaction after 1 year compared to usual care.

## Methods

### Trial design

This was a multicenter, open-label, parallel group study conducted in France (10 sites).

### Type of participants

Patients aged >12 and to <18 years with type 1 diabetes diagnosed at least 1 year earlier, with A1C rate >8 %, were eligible for our trial.

### Ethical approvals and consent

The study protocol was approved by the ethics committee “Comité de Protection des Personnes dans la Recherche Biomédicale Sud Est II” (file number 2005-100). All subjects and their parents provided informed consent before enrollment.

### Interventions

After central randomization participants were assigned to either an intervention group including a pediatrician visit every 3 months, a nurse visit intercalated every month and phone calls every 2 weeks after each visit; or to the control group with a pediatrician visit only every 3 months.

### Outcomes

The primary outcome was the evolution of the rate of A1C during the 12 months of follow-up.

Secondary outcomes were participants’ acceptance of the disease, evaluated by visual analog scale (VAS) (0 mm “I cope very well with my diabetes,” to 100 mm “I cope very badly with my diabetes”), the number of hypoglycemic or ketoacidosis episodes having required hospitalization, evaluation of A1C rate over time in each group, adolescents’, parents’ and nurses’ satisfactions evaluated by 6 questions..

### Randomization

Independent web-based platform according to a computer-generated randomization list and allowing concealed allocation was used to allocate participants to the intervention or control group. Randomization sequence was created using Stata (version 8, StataCorp, College Station, TX, USA) statistical software and was stratified by center with a 1:1 allocation using random block sizes of 4 and 6. Randomization was by a computer-generated random number list prepared by the department of biostatistics of the coordination center with no clinical involvement in the trial. After the physician had obtained the patient’s consent, he used the website for allocation consignment..

### Statistical analysis

For an absolute difference of decrease of the A1C at 12 months of 0.5 % between the 2 groups and considering a bilateral type I error of 5 %, a power of 80 % and a standard deviation of 1.5 % for a decrease of A1C at 12 months, 143 patients should be included in each group.

The primary outcome was the evolution of the rate of A1C during the follow-up, estimated using the measurements of A1C carried out every 3 months during 12 months in the adolescents of the 2 groups. A linear mixed model with random intercept and random slope was used to estimate this evolution taking into account intra-patient and inter- patient variability. The intervention effect was quantified by the difference of mean slope between the two groups. The difference of slope was estimated by the interaction parameter between the follow-up and the group. The parameters of the model were defined as significantly different from 0 for a *p* value < 0.05. The model was adjusted on three covariates, i.e. social environment defined as poor or rich, body mass index (BMI) and educational level (normal level or other level versus grade retention).

The same type of analysis was carried out to quantify the intervention effect on the patient’s acceptance of the disease measured every 3 months by a VAS.

The satisfaction of the adolescents and of the parents was described in the two groups without statistical comparison. It was also described for the nurses.

The SAS software version 9.1 (SAS Inc., Cary, NC, USA) was used for all analyses.

## Results

**Fig. 1:**
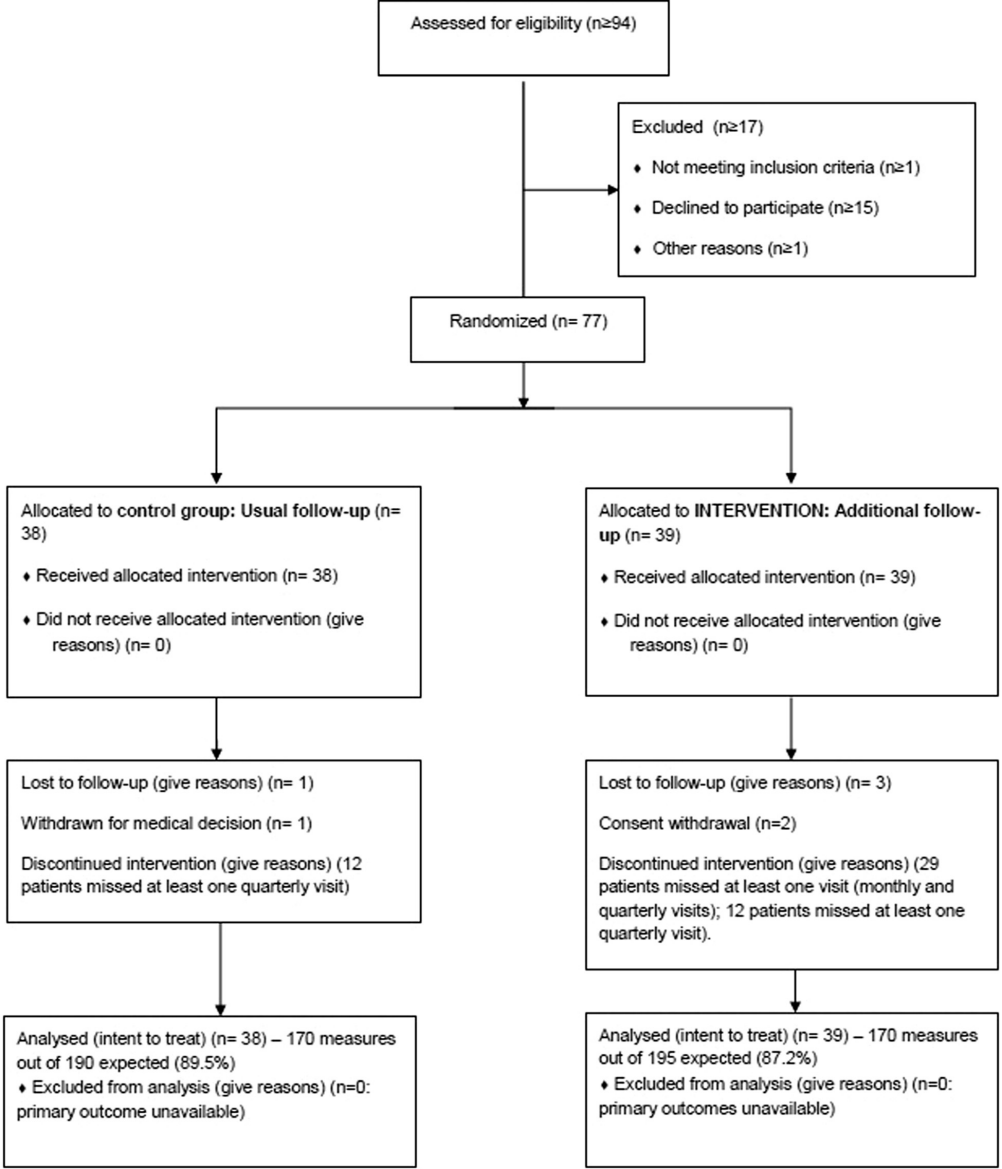
Flow diagram of patients

The flow diagram of patients is presented in Fig. 1. From March 2007 to August 2010, 77 patients, 38 in the control and 39 in the intervention group, were enrolled by 10 sites, 70 (90.9 %) completed the study, 2 withdrew their consent (2.6 %), 1 (2.6 %) was withdrawn because of the difficulties to follow study visits, and 4 (5.2 %) were lost from follow-up. Fifty-two adolescents participated in all quarterly visits. The total number of missed quarterly visits was 36 (11.7 %), 9.2 % (14) in the control and 14.1 % (22) in the intervention group. For nine patients A1C was measured by a local laboratory. Patients’ characteristics are shown in Table 1. At inclusion the mean rate of A1C was not significantly different between the control and the intervention group (10.1 % versus 10.2 %, *p* = 0.95) (Table 1). The acceptance of the disease measured at inclusion by a VAS between 0 (perfect acceptance) and 100 (very low acceptance), was also not significantly different between the control and the intervention groups (3.9 versus 4.9, *p* = 0.34).

**Table 1:** Participants’ characteristics

	Control	Intervention
	(*n* = 38)	(*n* = 39)
Age		
Mean (SD)	14.6 (1.6)	14.3 (1.6)
Range (min–max)	11.8–17.0	12.1–17.7
Gender		
Male *n* (%)	15 (39.5)	26 (66.7)
Female *n* (%)	23 (60.5)	13 (33.3)
Weight kg mean (SD)^a^	57.3 (13.5)	52.7 (12.7)
Height cm mean (SD)^a^	164 (9)	161 (11)
BMI mean (SD)	21.2 (3.6)	20.2 (3.3)
Social environment		
Poor *n* (%)	20 (52.6)	24 (61.5)
Rich *n* (%)	18 (47.4)	15 (38.5)
Educational level		
Grade retention *n* (%)	30 (79.0)	19 (48.7)
Normal *n* (%)	8 (21.1)	18 (46.2)
Other *n* (%)	0	2 (5.1)
A1C rate		
J0 % mean (SD)	10.1 (1.65)	10.2 (1.95)
T12–T0 % mean (SD)^a^	−0.18 (1.37)	−0.40 (1.26)
VAS (disease acceptance)		
J0 mean (SD)	3.9 (2.72)	4.9 (2.84)

^a^A1C was available for 36 patients in the control and 34 in the intervention group

*A1C* glycated hemoglobin, *BMI* body mass index, *SD* standard deviation, *VAS* visual analog scale

**Fig. 2:**
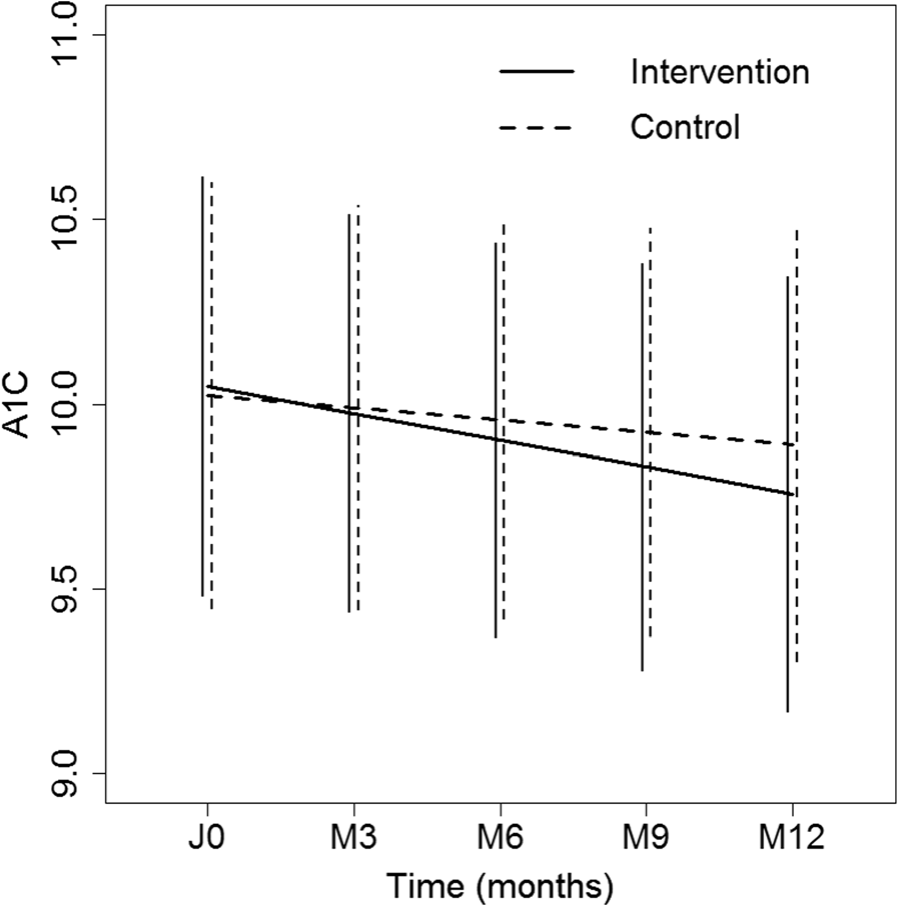
Evolution of A1C during the study in both groups (vertical line indicates 95% confidence intervals)

The primary analysis was in intention-to-treat and involved all patients who were randomly assigned. The decrease of the A1C rate in the control group was estimated at −0.03 % per month but this decrease was not statistically significant (95 % CI: (−0.14 %; 0.07 %), *p* = 0.54). The supplementary decrease of the A1C rate in the intervention group was estimated at −0.04 % (Fig. 2). However, this difference of slope was not statistically significant (95 % CI: (−0.19 %; 0.11 %), *p* = 0.61). The effect on the slope of social environment (rich versus poor: (−0.04 %, 95 % CI: (−0.20 %; 0.12 %), *p* = 0.62), of educational level (normal level versus grade retention: 0.015 %, 95 % CI: (−0.15 %; 0.18 %), *p* = 0.86; other level versus grade retention: −0.15 %, 95 % CI: (−0.62 %; 0.32 %), *p* = 0.53) and of BMI (−0.003 % per increase of 1 unit of BMI, 95 % CI: (−0.024 %; 0.017 %), *p* = 0.75) was not statistically significant. The adjustment on social environment, educational level and BMI did not modify the intervention effect on the slope.

**Fig. 3:**
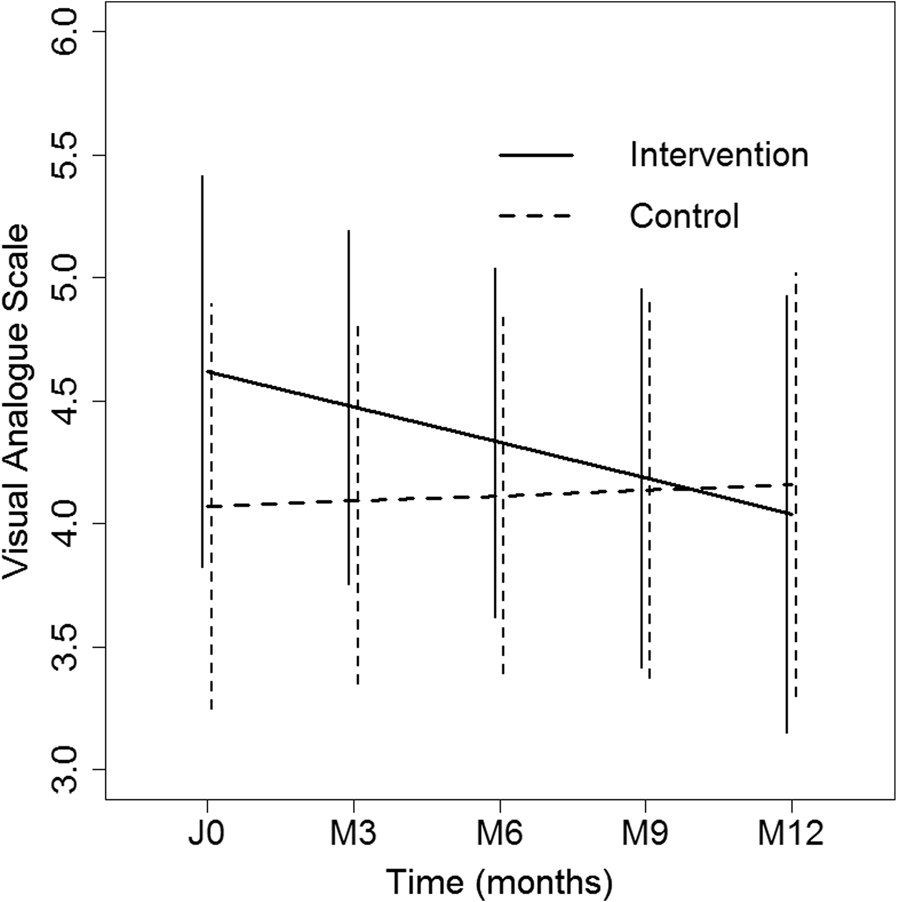
Evolution of participants' satisfaction in both groups during the study, measured by a VAS (from à “I cope very well with my diabetes” to 100 “I cope very badly with my diabetes” - Verticlal line indicates 95% confidence intervals)

For the disease acceptance, the slope quantifying the evolution of the mean VAS value was estimated at 0.022 per month in the control group and was not significantly different from 0 (95 % CI: (−0.19; 0.23), *p* = 0.83). The effect on the slope of the intervention was not statistically significant (−0.17, 95 % CI: (−0.47; 0.14), *p* = 0.28) (Fig. 3). The effect on the slope of social environment (rich versus poor: (−0.04, 95 % CI: (−0.47; 0.27), *p* = 0.81) and of BMI (0.003 per unit increase of BMI, 95 % CI: (−0.04; 0.04), *p* = 0.88) was not statistically significant. The effect on the slope of the educational level was estimated at −0.16 for the normal level versus grade retention (95 % CI: (−0.46; 0.14), *p* = 0.30) and at −0.87 for the other levels versus grade retention (95 % CI: (−1.69; −0.04), *p* = 0.04). The adjustment on social environment, educational level and BMI did not modify the intervention effect on the slope.

We also explored adolescents’, parents’ and nurses’ satisfactions. Adolescents’ and parent’s satisfaction are presented in Table 2. The satisfaction of nurses was explored by three questions. They found the dialog with the family and the therapeutic education very useful for eight (14.8 %, 19.2 % intervention versus 10.7 % control), useful for 32 (59.3 %), and not useful at all for 14 adolescents (25.9 %, 23.1 % intervention versus 28.6 % control). The time necessary for implementing the study was deemed acceptable for 40 (74.1 %, 76.9 % intervention versus 71.4 % control), very long for 9 (16.7 %, 23.1 % intervention versus 10.7 % control), and not very long for 5 (9.3 %) adolescents, all of them in the control group.

**Table 2:** Satisfaction of adolescents and parents

			Control	Intervention	Total
			*n* (%)	*n* (%)	*n* (%)
Were you satisfied by your global health management?	Very satisfied	Adolescents	6 (19.4)	4 (15.4)	10 (17.5)
		Parents	4 (14.3)	6 (24.0)	10 (18.9)
	Satisfied	Adolescents	21 (67.7)	21 (80.8)	42 (73.7)
		Parents	20 (71.4)	16 (64.0)	36 (67.9)
	Unsatisfied	Adolescents	2 (6.5)	1 (3.8)	3 (5.3)
		Parents	4 (14.3)	3 (12.0)	7 (13.2)
Do you feel that you now know better your diabetes better?	Yes	Adolescents	8 (25.8)	15 (57.7)	23 (40.4)
		Parents	11 (39.3)	11 (44.0)	22 (41.5)
	No	Adolescents	23 (74.2)	11 (42.3)	34 (59.6)
		Parents	17 (60.7)	14 (56.0)	31 (58.5)
Do you feel that you now manage your diabetes better?	Yes	Adolescents	13 (43.3)	20 (76.9)	33 (58.9)
		Parents	10 (35.7)	14 (56.0)	24 (45.3)
	No	Adolescents	17 (56.7)	6 (23.1)	23 (41.1)
		Parents	18 (64.3)	11 (44.0)	29 (54.7)
Did you spend a lot of time on the study?	A lot	Adolescents	2 (6.7)	3 (11.5)	5 (8.9)
	Not important	Adolescents	6 (20.0)	2 (7.7)	8 (14.3)
	Acceptable	Adolescents	20 (66.7)	20 (76.9)	40 (71.4)
	Inacceptable	Adolescents	2 (6.7)	1 (3.8)	3 (5.4)
Are you ready to continue?	Yes	Adolescents	19 (61.3)	18 (69.2)	37 (64.9)
	No	Adolescents	12 (38.7)	8 (30.8)	20 (35.1)
Efforts made for the study (time, travel and other commitments)	Very important	Parents	1 (3.7)	2 (8.0)	3 (5.8)
	Not important	Parents	9 (33.3)	4 (16.0)	13 (25.0)
	Acceptable	Parents	17 (63.0)	19 (76.0)	36 (69.2)

When they were asked whether nurse-counseling should be generalized for all children with type 1 diabetes, 44 (83.0 %) agreed and 9 (17.0 %) disagreed.

We did not detect any significant difference in the rate of adverse events between groups (Table 3), however, all adverse events (except ketoacidosis) were more common in the intervention group than in the control group.

**Table 3:** Number of adverse events reported in each group

	Control	Intervention	Total
Metabolic and nutritional disorders			
Acetonemia	0	1	1
Diabetes mellitus inadequately controlled	8	10	18
Diabetic ketoacidosis	5	7	12
Hypoglycemia	1	2	3
Hypoglycemic seizure	0	1	1
Insulin hypoglycemia	0	1	1
Hyperglycemia	0	2	2
Ketoacidosis	1	0	1
Social circumstances			
Investigations	1	0	1
Social stay hospitalization	1	0	1
Treatment non-compliance	0	1	1
Nervous system disorders	3	2	5
Psychiatric disorders	1	0	1
Surgical and medical procedures	5	1	6
Total	26	28	54

## Discussion

To our knowledge this is the first randomized controlled trial comparing nurse-counseling to usual care in order to improve A1C control in adolescents with type 1 diabetes and poor metabolic control. Our results did not show any significant effect in terms of A1C control or quality of life of adolescents after 1 year follow-up. Adherence seems to have an important role for glycemic control in type 1 diabetes of adolescents [4]. In our study, adherence to the protocol was low because only 67.5 % of adolescents participated in all visits. However, the total number of missed visits was not significantly different between groups and tended to be lower in the intervention group (9.9 % intervention versus 14.1 % control). Our results also show that nurse satisfaction was high and that they were keen to generalize the program. Adolescents seemed to know (57.7 % versus 25.8 %), and manage their diabetes better (76.9 % versus 43.3 %) in the intervention group compared with the control group, although the differences were not statistically significant. Parents seemed also to manage the diabetes of their child better in the intervention group (56.0 % versus 35.7 %) but their knowledge had not been significantly modified (44.0 % versus 39.3 %).

Regarding the adverse events rate, the trend highlighted might be due to patients feeling that receiving an intervention will improve their glycemic control by default.

Filling the gap between physician’s knowledge and patients’, reinforced management by nurses has shown a positive effect in the management of hypertension in patient with diabetes [14] and seems cost-effective [15]. A large study on Medicare adult patients with congestive heart failure, coronary artery disease and diabetes [16], however, failed to show any benefit on hospitalization rate in 13 of the 15 hospitals involved. Systematic reviews of the literature on non-pharmacological intervention in type 1 diabetes in children and adolescents also show that only psychological interventions and management by telemedicine slightly but significantly decrease the A1C [12, 17–20]. The positive effect on A1C seemed to be confirmed by a French program (called Sophia) including 100,000 diabetic patients followed-up by phone by nurses giving appropriate advice. According to preliminary results after 3 years, it seems that this population has better follow-up (is more systematically undergoing specific exams like laboratory analysis, ophthalmologic or cardiologic examination), has better diabetes control (A1C improvement) and that this strategy is economically relevant. The presence of selection bias and regression to the mean can, however, affect this positive result [21].

The adolescents included in our study had a higher A1C at baseline (10 %) compared to the A1C reported in adolescents during a summer camp in France (8 %) [9]. During the trial most centers had already implemented nurse-counseling and randomizing patients to a control group without intervention was felt to be unethical leading to the enrolment of those adolescents that are very poorly controlled.

Because of difficulties encountered in enrolling patients our study was stopped before reaching the target population of 300 patients. As a result, our study is not powered enough to conclude on the primary outcome. Our results show, however, consistent trends in favor of the intervention on the change in A1C on these patients.

## Conclusion

Public health decisions should be based on relevant unbiased evidence provided by comparative studies. Unjustified enthusiasm and premature implementation of costly educational or interventional programs make their adequate evaluation prior to making decisions difficult.

More studies are needed in order to show whether nurse-led intervention might improve the management of diabetes and its clinical consequences in adolescents.

## Abbreviations

A1C: glycated hemoglobin
BMI: body mass index
CI: confidence interval
SD: standard deviation
VAS: visual analog scale
